# Longitudinal SARS-CoV-2 humoral response in MS patients with and without SARS-CoV-2 infection prior to vaccination

**DOI:** 10.3389/fneur.2022.1032830

**Published:** 2022-11-10

**Authors:** Koos P. J. van Dam, Laura Hogenboom, Eileen W. Stalman, Laura Y. L. Kummer, Maurice Steenhuis, Jim B. D. Keijser, Anja ten Brinke, S. Marieke van Ham, Taco W. Kuijpers, Theo Rispens, Luuk Wieske, Filip Eftimov, Eva M. Strijbis, Joep Killestein, Zoé L. E. van Kempen

**Affiliations:** ^1^Department of Neurology and Neurophysiology, Amsterdam Neuroscience, Amsterdam UMC, Location AMC, University of Amsterdam, Amsterdam, Netherlands; ^2^Department of Neurology, Amsterdam UMC, Vrije Universiteit, Amsterdam, Netherlands; ^3^Department of Immunopathology, Sanquin Research and Landsteiner Laboratory, Amsterdam, Netherlands; ^4^Biologics Laboratory, Sanquin Diagnostic Services, Amsterdam, Netherlands; ^5^Swammerdam Institute for Life Sciences, University of Amsterdam, Amsterdam, Netherlands; ^6^Department of Pediatric Immunology, Rheumatology and Infectious Disease, Amsterdam UMC, Location AMC, Emma Children's Hospital, University of Amsterdam, Amsterdam, Netherlands; ^7^Department of Clinical Neurophysiology, St. Antonius Hospital, Nieuwegein, Netherlands

**Keywords:** multiple sclerosis, SARS-CoV-2, COVID-19, disease modifying treatment, humoral response

## Abstract

**Introduction:**

During the COVID-19 pandemic, certain disease modifying therapies (DMTs) used in multiple sclerosis (MS), such as anti-CD20 therapies, have been associated with decreased humoral responses after SARS-CoV-2 vaccination. Hybrid immunity, referring to immunity after both vaccination and SARS-CoV-2 infection might increase humoral responses.

**Methods:**

This was a substudy of two prospective cohort studies on SARS-CoV-2 antibodies after SARS-CoV-2 infection and vaccination. RBD-specific IgG titers of patients with MS and healthy controls who had experienced SARS-CoV-2 infection prior to the first vaccination were compared with those patients and healthy controls without prior infection. Humoral responses were measured at various time points after SARS-CoV-2 infection in convalescent patients and all patients prior to the first vaccination, 28 days after the first vaccination, and 28 days after the second vaccination.

**Results:**

One hundred and two individuals [of which 34 patients with MS and DMTs (natalizumab or ocrelizumab), 30 patients without DMTs, and 38 healthy controls] were included. Fifty one of these individuals were convalescent. Median SARS-CoV-2 antibody titers were higher after the first vaccination in convalescent individuals compared with individuals without infection prior to vaccination. Severe acute respiratory syndrome coronavirus 2 (SARS-CoV-2) antibody titers were comparable after the second vaccination in patients with MS with and without prior infection. However, in the convalescent ocrelizumab-treated patients, SARS-CoV-2 antibody titers did not increase after vaccinations.

**Conclusion:**

In patients with MS without anti-CD20 therapies, SARS-CoV-2 infection before vaccination increases humoral responses after the first vaccination, similar to the healthy controls. In patients with MS treated with ocrelizumab (convalescent and non-convalescent), humoral responses remained low.

## Introduction

Since the start of the COVID-19 pandemic, humoral, and cellular immunity against SARS-CoV-2 antigen has been extensively studied after vaccinations. Within the population with multiple sclerosis (MS), anti-CD20 therapies (e.g., ocrelizumab and rituximab) were shown to severely impair humoral responses after SARS-CoV-2 vaccination (1). In patients with MS without disease modifying therapies (DMTs) or patients with MS on non-immunosuppressive DMTs, immunity after vaccination is largely comparable to healthy controls (2).

Despite vaccination, infection with SARS-CoV-2 can result in breakthrough COVID-19, even though vaccination is effective in preventing severe COVID-19. Hybrid immunity, resulting from SARS-CoV-2 infection and vaccination combined, has been shown to increase potency and breadth of SARS-CoV-2 antibodies in healthy individuals (3). The observation that SARS-CoV-2 breakthrough infections are more frequent among patients with MS and low SARS-CoV-2 antibody titers (4), gives rise to the question of whether hybrid immunity in MS leads to a better humoral immune response and clinical protection than vaccination only. In patients with MS, data are scarce regarding the effects of hybrid immunity on humoral responses and SARS-CoV-2 breakthrough infections.

The objective of this study was to evaluate the humoral immune response and SARS-CoV-2 breakthrough infections in patients with MS and healthy controls with and without SARS-CoV-2 infection prior to the first vaccination.

## Materials and methods

From August to December 2020, before the availability of SARS-CoV-2 vaccinations, patients with MS from the Amsterdam MS Center, the Netherlands, were tested for SARS-CoV-2 antibodies (COMS-19 study, ClinicalTrials.gov Identifier: NCT04498286) (5). Patients with a positive SARS-CoV-2 antibody response or a positive PCR prior to vaccination were longitudinally followed in another prospective cohort study on vaccination against SARS-CoV-2 in patients with various immune-mediated inflammatory diseases (T2B!; Trial NL8900; Dutch Trial register) (6).

For this substudy, patients with MS treated with ocrelizumab, natalizumab, or no DMTs who have had a SARS-CoV-2 infection (defined by positive PCR and/or positive SARS-CoV-2 antibodies) prior to the first vaccination were included. Matched controls without prior SARS-CoV-2 infection from the T2B! study were included (1:1) matching for DMT, age, and sex. Furthermore, a group of healthy controls with and without SARS-CoV-2 infection prior to vaccination was included. All patients in the SARS-CoV-2 negative control groups were tested negative for SARS-CoV-2 antibodies at baseline (prior to the first vaccination).

Clinical data and data regarding SARS-CoV-2 (breakthrough) infections were retrieved from the medical files and electronic questionnaires, which were sent to patients every 2 months after the first vaccination. When a patient indicated a positive PCR or antigen test, that patient was contacted by a researcher at least 2 weeks after the positive test to verify and determine COVID-19 severity. Coronavirus disease (COVID-19) severity was based on the WHO classification.

In the COMS-19 study, serum samples were cross-sectionally collected by venipuncture in a large cohort of patients with MS and variable timing of sampling since SARS-CoV-2 infection and until the first vaccination. For follow-up in the T2B! study, serum samples were collected by venipuncture or by participants at home using a finger prick set. Samples were taken at predefined time points: at baseline (prior to the first vaccination) and day 28 after the first and second vaccination (when applicable). Serum was not available for all patients at all time points (see Table 1).

**Table 1:** Baseline characteristics and data on humoral responses.

	**Natalizumab** **(n: 18)**		**Ocrelizumab** **(n: 16)**		**No DMT** **(n: 30)**		**Healthy controls** **(n: 38)**	
**SARS-CoV-2 infection prior to vaccination, n (%)**	**Yes**, **9 (50)**	**No**, **9 (50)**	**Yes**, **8 (50)**	**No**, **8 (50)**	**Yes**, **15 (50)**	**No**, **15 (50)**	**Yes**, **19 (50)**	**No**, **19 (50)**
Age, mean (SD)	44 (12)	46 (8)	50 (10)	53 (3)	55 (9)	55 (3)	55 (3)	55 (4)
Female sex, *n* (%)	8 (89)	8 (89)	4 (50)	4 (50)	10 (67)	10 (67)	14 (74)	14 (74)
**COVID-19 severity prior to vaccination, n (%)**								
Asymptomatic	2 (33)	NA	3 (38)	NA	2 (13)	NA	3 (18)	NA
Mild	7 (78)	NA	4 (50)	NA	13 (87)	NA	14 (82)	NA
Moderate	0	NA	0	NA	0	NA	0	NA
Severe	0	NA	1 (13)	NA	0	NA	0	NA
**Vaccination type primary immunization, n (%)**								
AstraZeneca	1 (11)	1 (11)	1 (13)	0	4 (27)	0	0	0
Janssen	0	0	1 (13)	0	0	0	0	0
Moderna	2 (22)	2 (22)	3 (38)	7 (89)	3 (20)	8 (53)	13 (69)	13 (69)
Pfizer/BioNtech	6 (67)	6 (67)	3 (38)	1 (13)	8 (53)	7 (47)	6 (32)	6 (32)
**Anti-RBD titer** **>4 AU/mL prior to first vaccination, n (%)**								
Seroconversion	5 (63)	0	4 (67)	0	6 (75)	0	11 (61)	0
No seroconversion	3 (38)	9 (100)	2 (33)	7 (100)	2 (25)	15 (100)	7 (39)	18 (100)
Serology missing	0	0	2	1	7	0	1	1
**Anti-RBD titer prior to first vaccination, median (IQR)**								
Titer	7.5 (2.3–16.9)	NA	8.4 (0.7–31.8)	NA	6.2 (3.4–9.7)	NA	4.42 (1.2–13.0)	NA
**Anti-RBD titer** **>4 AU/mL 28 days after 1st vaccination, n (%)**								
Seroconversion	6 (100)	6 (67)	3 (50)	1 (14)	14 (93)	14 (93)	17 (100)	18 (100)
No seroconversion	0	3 (33)	3 (50)	6 (86)	1 (7)	1 (7)	0	0
Serology missing	3	0	2	1	0	0	2	1
**Anti-RBD titer 28 days after 1st vaccination, median (IQR)**								
Titer	180.0 (54.4–271.0)	12.9 (3.0–36.9)	11.7 (1.0–38.5)	0.5 (0.1–1.6)	163.0 (21.6–545.0)	23.3 (10.9–48.9)	300.0 (218.0–558.0)	22.9 (12.2–34.1)
**Anti-RBD titer** **>4 AU/mL 28 days after 2nd vaccination, n (%)**								
Seroconversion	9 (100)	9 (100)	2 (33)	3 (38)	14 (100)	14 (100)	15 (100)	19 (100)
No seroconversion	0	0	4 (67)	5 (63)	0	0	0	0
Serology missing	0	0	2	0	1	1	4	0
**Anti-RBD titer 28 days after 2nd vaccination, median (IQR)**								
Titer	205.0 (158.0–341.0)	135 (72.9–662)	0.6 (0.1–12.7)	1.0 (0.1–12.3)	188.0 (83.0–329.0)	157.0 (69.8–250.0)	356.0 (237.0–662.0)	228.0 (88.9–289.0)
**Breakthrough infection in 180 days after second vaccination, n (%)**								
SARS-CoV-2 infection	0 (0)	0 (0)	0 (0)	1 (13)	0 (0)	1 (7)	0 (0)	1 (5)

Baseline characteristics and humoral data in patients categories with SARS-CoV-2 infection prior to the primary vaccination and matched controls without prior SARS-CoV-2 infection. In two healthy controls, severity of COVID-19 is missing. Seroconversion after vaccination was defined as antibody titers >4 AU/mL. The median time in days between last ocrelizumab infusion and first vaccination was 116 (IQR 25–157) in convalescent patients and 136 (IQR 20–152) in non-convalescent ocrelizumab-treated patients.

The serum was assessed using a quantitative anti-RBD IgG enzyme-linked immunosorbent assay (ELISA), as described previously (7). Anti-RBD IgG titers were expressed as arbitrary units (AU) per mL (AU/mL) and were compared with a serially diluted calibrator (arbitrarily assigned a value of 100 AU/mL) consisting of pooled convalescent plasma. Seroconversion after vaccination was defined as antibody titers >4 AU/mL.

Differences in proportions were analyzed using Fisher's exact test, and differences between continuous variables were analyzed using the Wilcoxon rank-sum test. Analyses were performed in R version 4.2.1 (R Foundation for Statistical Computing, Vienna, Austria).

## Results

**Figure 1:**
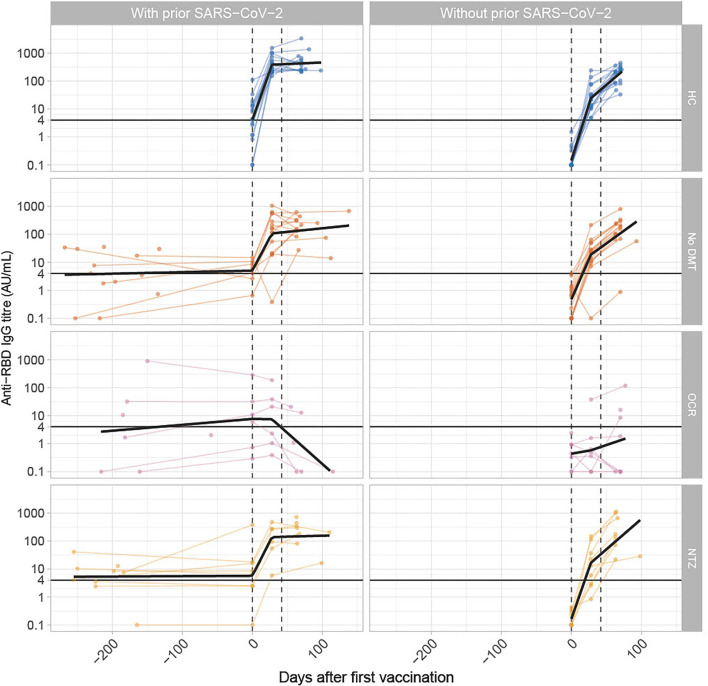
Longitudinal SARS-CoV-2 antibody titers in patients with and without prior SARS-CoV-2 infection. The figure shows anti-RBD titers over time in patients with MS in various treatment groups. The x-axis shows time in days before and after the first vaccination. The y-axis is a logarithmic scale of anti-RBD-IgG titers in AU/mL. The Dots indicate antibody titers for individual participants. The black line indicates the regression line per group to demonstrate a trend. The horizontal black line indicates the cut-off for seroconversion (>4 AU/mL). The dotted lines, respectively, indicate the median timing of the first and second vaccination, either with mRNA vaccines (Moderna/Pfizer) or ChAdOx1 nCoV-19 (AZD1222) vaccine from Oxford-AstraZeneca. The median time between the first and second vaccination was 42 days.

One hundred and two participants were included in this substudy, of which 34 patients with MS and DMTs (natalizumab or ocrelizumab), 30 patients without DMTs, and 38 healthy controls. Baseline characteristics and humoral responses of participants with (*n* = 51) and without prior SARS-CoV-2 infection (*n* = 51) are described in the Table 1. Longitudinal results of SARS-CoV-2 anti-RBD antibody titers are presented in the Figure 1.

The exact time of SARS-CoV-2 infection prior to vaccination was available in 47% (24/51) of participants as PCR testing was not widely available in the Netherlands in early 2020. The median time from positive PCR to first vaccination in patients with available data was 229 days (IQR 175–304). In remaining patients with MS without a date of SARS-CoV-2 infection, infection occurred prior to first sampling which took place a median of 216 days (IQR 185–231) prior to the first vaccination.

Before the first vaccination, 65% (26/40) of all participants with prior SARS-CoV-2 infection had anti-RBD IgG titers above the threshold of 4 AU/mL; however, titers were low [median titer 10.9 AU/mL (IQR 6.5–17.8)].

At day 28 after the first vaccination, 85% (23/27) of patients with MS and prior SARS-CoV-2 infection had anti-RBD IgG titers above the threshold of 4 AU/mL vs. 68% (21/31) of patients with MS without SARS-CoV-2 infection (*p*: 0.14, Table 1). In patients with MS on natalizumab, patients without DMTs, and healthy controls, the anti-RBD antibody titer after the first vaccination was higher in participants with prior SARS-CoV-2 infection than in participants without prior infection to vaccination (*p*: 0.04, *p* < 0.01, and *p* < 0.001, respectively, Table 1). In contrast, no difference was identified in anti-RBD titers after the first vaccination in patients with MS on ocrelizumab with or without a prior SARS-CoV-2 infection (*p*: 0.07, Table 1).

At day 28 after the second vaccination, all individuals were seroconverted (anti-RBD titer >4 AU/mL 28 days after the second vaccination), with an exception of ocrelizumab-treated patients (36%, 5/14). Antibody titers in patients with MS on natalizumab, without DMTs, and healthy controls, were all higher after the second vaccination compared with the first vaccination (Table 1). In patients on natalizumab and without DMTs, no significant differences in titer were observed in patients with and without prior SARS-CoV-2 infection (*p*: 0.73 and *p*: 0.48, respectively, Table 1), whereas for healthy controls, the median anti-RBD antibody was higher by 1.6-fold (*p* < 0.01, Table 1). In ocrelizumab-treated patients, seroconversion and anti-RBD antibody titers remained low after vaccinations, also in the group with prior SARS-CoV-2 infection.

SARS-CoV-2 breakthrough infections in 6 months (180 days) after the second vaccination were reported by 3% (3/102) of participants, all females without prior SARS-CoV-2 infection. These breakthrough infections occurred in one 53 years old healthy control who had mild symptomatic disease (no assistance needed), one 50 years old patient without DMT who had mild symptomatic disease (no assistance needed), and one 50 years old ocrelizumab-treated patient who had moderate disease (hospitalization with oxygen). The patient on ocrelizumab was hospitalized for supportive treatment including oxygen suppletion for 10 days after which she recovered and was discharged.

## Discussion

In this study, in patients with MS on natalizumab and without DMTs and healthy controls with prior SARS-CoV-2 infection, higher SARS-CoV-2 antibody titers were found 28 days after the first vaccination compared with matched individuals without SARS-CoV-2 infection. In these patients, further increase of anti-RBD titer was limited after the second vaccination. Our results are in agreement with Shenoy et al. who reported an increase in antibody titers after vaccination in convalescent patients with autoimmune rheumatic diseases compared with non-convalescent patients (8). In this study, part of the patients who experienced COVID-19 did not show seroconversion when tested prior to vaccination. As immunity and seroconversion after infection or vaccination against SARS-CoV-2 wanes over time, a full vaccination cycle is also recommended following international guidelines in patients with MS andprior SARS-CoV-2 infection. Our data show no difference in SARS-CoV-2 antibody titers after a full vaccination cycle between convalescent and non-convalescent patients with MS. Therefore, patients with MS and hybrid immunity (including a full vaccination cycle) might have comparable immunity compared with non-convalescent patients, although the potency and breadth of SARS-CoV-2 antibodies could be different after vaccination or infection, which was not evaluated in this current study.

In ocrelizumab-treated patients, we found low seroconversion rates and anti-RBD antibody titers in patients with and without prior SARS-CoV-2 infection. Anti-CD20 therapies greatly impair the antibody response after SARS-CoV-2 infection and vaccination in patients with MS (1, 9). Therefore, patients are offered additional vaccinations to increase humoral responses. The majority of these patients, however, remain seronegative after the third vaccination (10). Decreased humoral responses are shown to increase the risk of SARS-CoV-2 breakthrough infection, but severe breakthrough infection is rare likely due to intact T cell responses (4).

Our study has limitations, the most important one being the limited sample size. In addition, we were unable to study patients on sphingosine 1-phosphate receptor modulators, a therapy known to inhibit the humoral and cellular responses after SARS-CoV-2 vaccination, as only five patients on fingolimod with a SARS-CoV-2 infection were identified in the COMS-19 study who did not complete follow-up after vaccination (5). Another limitation was missing data regarding the timing of COVID-19 prior to vaccination in half of our patients, as the timing influences antibody titers and level of immunity. Furthermore, our results might not be translatable to later variants of SARS-CoV-2. The strength of this study was the prospective design and sampling at pre-specified time points after vaccination.

In conclusion, prior SARS-CoV-2 infection increases anti-RBD antibody responses after the first vaccination in patients with MS. However, in ocrelizumab-treated patients, humoral responses remained low and also in convalescent participants.

## Data availability statement

The raw data supporting the conclusions of this article will be made available by the authors, without undue reservation.

## Ethics statement

The studies involving human participants were reviewed and approved by Medical Ethical Committee of the VU University Medical Center/Medical Ethical Committee of the Amsterdam Medical Center. The patients/participants provided their written informed consent to participate in this study.

## Author contributions

ZK, KD, TK, AB, SH, TR, FE, and JKi contributed to the conception and design of the study. ZK, LH, EWS, EMS, MS, LK, KD, Jke, and LW contributed to the acquisition and analysis of the data. KD performed the statistical analyses. ZK and KD drafted a significant portion of the manuscript or figures. All authors revised the manuscript critically for intellectual content. All authors contributed to the article and approved the submitted version.

## Funding

We are very grateful to the Dutch MS Research Foundation for funding the COMS-19 study (grant 20-005 PP) and for ZonMw (The Netherlands Organization for Health Research and Development) for funding the T2B! study (Grant No. 10430072010007). Both sponsors had no role in the design, analyses, or reporting of the study.

## Conflict of interest

FE reports consulting fees from UCB Pharma and CSl Behring; honoraria from Grifols. TR reports consulting fees from Novartis. Jki reported speaking and consulting relationships with Biogen, Genzyme, Merck, Novartis, Roche, Sanofi, and TEVA. Amsterdam UMC, location VUmc, MS Center Amsterdam has received financial support for research activities from Biogen, Celgene, Genzyme, Merck, Novartis, Roche, Sanofi, and TEVA. The remaining authors declare that the research was conducted in the absence of any commercial or financial relationships that could be construed as a potential conflict of interest.

## Publisher's note

All claims expressed in this article are solely those of the authors and do not necessarily represent those of their affiliated organizations, or those of the publisher, the editors and the reviewers. Any product that may be evaluated in this article, or claim that may be made by its manufacturer, is not guaranteed or endorsed by the publisher.
